# Neurosecretory Protein GL Accelerates Liver Steatosis in Mice Fed Medium-Fat/Medium-Fructose Diet

**DOI:** 10.3390/ijms23042071

**Published:** 2022-02-13

**Authors:** Yuki Narimatsu, Eiko Iwakoshi-Ukena, Mana Naito, Shogo Moriwaki, Megumi Furumitsu, Kazuyoshi Ukena

**Affiliations:** Laboratory of Neurometabolism, Graduate School of Integrated Sciences for Life, Hiroshima University, Higashi-Hiroshima, Hiroshima 739-8521, Japan; d214243@hiroshima-u.ac.jp (Y.N.); iwakoshi@hiroshima-u.ac.jp (E.I.-U.); m205336@hiroshima-u.ac.jp (M.N.); m203300@hiroshima-u.ac.jp (S.M.); mfurumi@hiroshima-u.ac.jp (M.F.)

**Keywords:** neurosecretory protein GL, hypothalamus, neuropeptide, obesity, dietary sugar, liver steatosis

## Abstract

Sugar consumption can readily lead to obesity and metabolic diseases such as liver steatosis. We previously demonstrated that a novel hypothalamic neuropeptide, neurosecretory protein GL (NPGL), promotes fat accumulation due to the ingestion of sugar by rats. However, differences in lipogenic efficiency of sugar types by NPGL remain unclear. The present study aimed to elucidate the obesogenic effects of NPGL on mice fed different sugars (i.e., sucrose or fructose). We overexpressed the NPGL-precursor gene (*Npgl*) in the hypothalamus of mice fed a medium-fat/medium-sucrose diet (MFSD) or a medium-fat/medium-fructose diet (MFFD). Food intake and body mass were measured for 28 days. Body composition and mRNA expression of lipid metabolic factors were measured at the endpoint. *Npgl* overexpression potently increased body mass with fat accumulation in the white adipose tissue of mice fed MFFD, although it did not markedly affect food intake. In contrast, we observed profound fat deposition in the livers of mice fed MFFD but not MFSD. In the liver, the mRNA expression of glucose and lipid metabolic factors was affected in mice fed MFFD. Hence, NPGL induced liver steatosis in mice fed a fructose-rich diet.

## 1. Introduction

Diets with unbalanced nutrients lead to obesity and ectopic fat deposition, resulting in metabolic syndrome [1,2]. Recent studies have revealed that the ingestion of sugars, such as sucrose and fructose, profoundly aggravates type 2 diabetes, fatty liver, and hypertension due to overnutrition and changes in systemic energy metabolism [3,4,5]. Thus, detailed research focusing on the relationship between dietary sugar and energy metabolism is required to treat metabolic syndromes.

Several studies have demonstrated that systemic energy metabolism is controlled by hypothalamic neuropeptides and peripheral hormones. The arcuate nucleus in the hypothalamus, which regulates feeding and energy metabolism, produces potent feeding-related neuropeptides. For instance, neuropeptide Y (NPY) and agouti-related peptide (AgRP) are well-known orexigenic neuropeptides [6,7,8]. Conversely, α-melanocyte-stimulating hormone (α-MSH), derived from proopiomelanocortin (POMC), functions as an anorexigenic neuropeptide via melanocortin receptor type 4 (MC4R) [9]. In contrast, leptin and insulin are peripheral hormones involved in energy metabolism. Leptin is secreted by white adipose tissue (WAT) and acts as an anorexigenic hormone by inhibiting the activity of NPY/AgRP neurons [10]. Insulin stimulates lipid deposition by inhibiting lipolysis [11]. In addition, a few studies have reported that neuropeptides and hormones manipulate feeding behavior and energy metabolism depending on the nutritional composition of diets, especially carbohydrates. Corticotropin-releasing hormone (CRH) exerts palatable effects on carbohydrates in mice [12], whereas fibroblast growth factor 21 (FGF21) inhibits sugar intake [13]. Although there is accumulating evidence, which suggests that energy metabolic regulation via the endocrine system participates in the intake of carbohydrates, there are few observations concerning the relationships between types of sugar consumed and the endocrine system.

To understand the mechanisms involved in energy metabolism, we recently identified a novel gene from the hypothalamus of chickens, rats, mice, and humans [14,15,16]. Since the novel gene produces a small secretory protein whose C-terminus amino acid sequence is Gly-Leu-NH_2_, it was termed neurosecretory protein GL (NPGL) [14]. The primary structure of NPGL is evolutionarily conserved in vertebrates, although the receptor for NPGL has not yet been discovered [17]. To reveal the physiological function of NPGL, we observed its effects on chickens, rats, and mice. Chronic administration of NPGL enhanced lipid metabolism in chickens [18]. Furthermore, NPGL promoted the intake of carbohydrate and fat accumulation via de novo lipogenesis in rats [15,19]. Moreover, overexpression of the NPGL-precursor gene (*Npgl*) rapidly induced obesity in mice [20]. Thus, we speculate that NPGL exerts obesogenic effects by evoking the palatability of sugars in birds and mammals. Although sucrose and fructose are well-known as representative sugars in human nutrition [5], the sugars that are easily used in lipogenesis by NPGL remain unclear.

To reveal the lipogenic efficiency of NPGL with different types of sugar, we induced hypothalamic overexpression of *Npgl* in mice fed a medium-fat/medium-sucrose diet (MFSD) or a medium-fat/medium-fructose diet (MFFD). Here, we report that more potent liver steatosis was observed in *Npgl* overexpressed mice fed MFFD than in mice fed MFSD. This study reveals the effects of *Npgl* overexpression on food intake, body mass, body composition, and blood parameters in mice.

## 2. Results

### 2.1. Effects of NPGL-Precursor Gene Overexpression on Food Intake, Body Mass, and Food Efficiency

**Figure 1:**
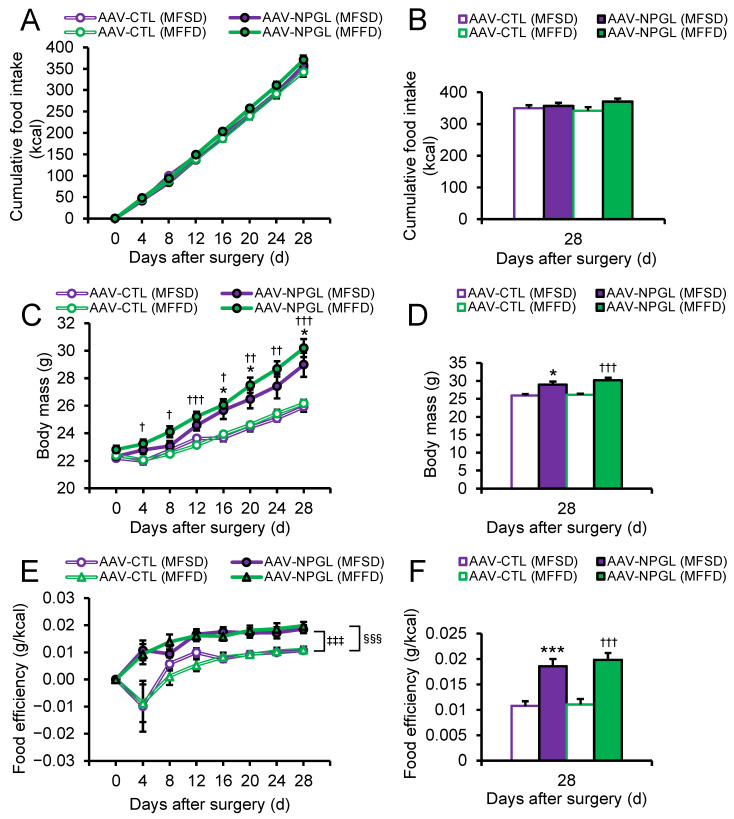
Effects of *Npgl* overexpression on food intake, body mass, and food efficiency. The panels show the data obtained after injection of AAV-CTL or AAV-NPGL into mice fed MFSD or MFFD for 28 days. (**A**) Cumulative food intake at all points. (**B**) Cumulative food intake 28 days after injection. (**C**) Body mass at all points. (**D**) Body mass 28 days after injection. (**E**) Food efficiency expressed as body weight gain per cumulative food intake per week at all points. (**F**) Food efficiency 28 days after injection. Each value represents the mean ± standard error of the mean (*n* = 5–6/group). * *p* < 0.05, *** *p* < 0.005 AAV-CTL (MFSD) vs. AAV-NPGL (MFSD) in the same period by one-way ANOVA with Tukey’s test for multiple comparisons, ^†^ *p* < 0.05, ^††^ *p* < 0.01, ^†††^ *p* < 0.005 AAV-CTL (MFFD) vs. AAV-NPGL (MFFD) in the same period by one-way ANOVA with Tukey’s test for multiple comparisons, ^‡‡‡^ *p* < 0.005 AAV-CTL (MFSD) vs. AAV-NPGL (MFSD) by two-way ANOVA by repeated measures with Bonferroni’s test for multiple comparisons, ^§§§^ *p* < 0.005 AAV-CTL (MFFD) vs. AAV-NPGL (MFFD) by two-way ANOVA by repeated measures with Bonferroni’s test for multiple comparisons. NPGL, neurosecretory protein GL; AAV-CTL, AAV-based control vector; AAV-NPGL, AAV-based NPGL-precursor gene vector; MFSD, medium-fat/medium-sucrose diet; MFFD, medium-fat/medium-fructose diet.

To reveal the effects of NPGL on lipid metabolism attributed to the different types of sugars, we overexpressed *Npgl* using adeno-associated virus (AAV) in mice fed MFSD or MFFD. Two-way repeated-measures ANOVA showed the main effect of time and time × group interaction on food intake (Figure 1A). One-way ANOVA revealed that food intake was not affected by *Npgl* overexpression in mice (Figure 1B). The main effects of time, group, and time × group interaction were significant on body mass as indicated by two-way repeated-measures ANOVA (Figure 1C). The effects of *Npgl* overexpression on body mass gain were observed at early phases (day 4 after surgery) in mice fed MFFD (Figure 1C). One-way ANOVA showed that body mass was increased by *Npgl* overexpression in mice fed the two diets (Figure 1D). We subsequently calculated food efficiency, which is an index of the amount of mass gained per unit of food intake [20]. Two-way ANOVA with repeated measures revealed the main effects of time and treatment on food efficiency (Figure 1E). Bonferroni’s test showed that food efficiency was increased by *Npgl* overexpression under the two diets (Figure 1E). Food efficiency was significantly augmented by *Npgl* overexpression at the endpoint (Figure 1F). Based on these data, *Npgl* overexpression stimulated body mass gain without changing food intake in mice, especially when fed MFFD.

### 2.2. Effects of NPGL-Precursor Gene Overexpression on Body Composition and Serum Parameters

**Figure 2:**
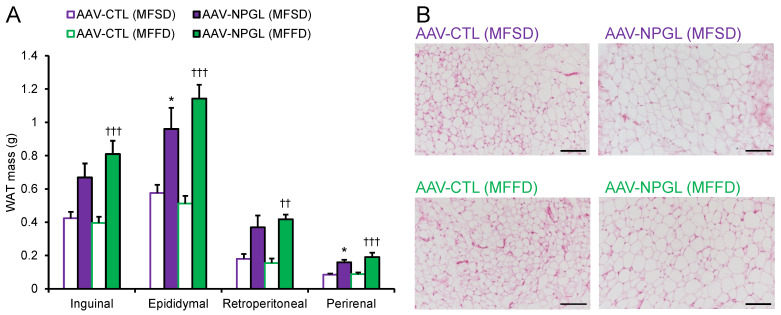
Effects of *Npgl* overexpression on fat accumulation. The panels show the data obtained after injection of AAV-CTL or AAV-NPGL into mice fed MFSD or MFFD for 28 days. (**A**) Mass of the inguinal, epididymal, retroperitoneal, and perirenal WAT. (**B**) Representative images of sections of the inguinal WAT of mice fed MFSD or MFFD. Scale bars = 100 µm. Each value represents the mean ± standard error of the mean (*n* = 5–6). Differences between groups were assessed by one-way ANOVA with Tukey’s test for multiple comparisons. * *p* < 0.05 AAV-CTL (MFSD) vs. AAV-NPGL (MFSD), ^††^ *p* < 0.01, ^†††^ *p* < 0.005 AAV-CTL (MFFD) vs. AAV-NPGL (MFFD). NPGL, neurosecretory protein GL; AAV-CTL, AAV-based control vector; AAV-NPGL, AAV-based NPGL-precursor gene vector; MFSD, medium-fat/medium-sucrose diet; MFFD, medium-fat/medium-fructose diet; WAT, white adipose tissue.

**Figure 3:**
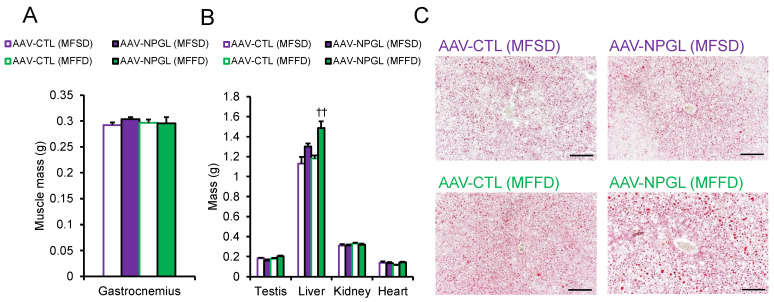
Effects of *Npgl* overexpression on muscles and organs. The panels show the data obtained after injection of AAV-CTL or AAV-NPGL into mice fed MFSD or MFFD for 28 days. (**A**) Mass of the gastrocnemius muscle. (**B**) Mass of the testis, liver, kidney, and heart. (**C**) Representative liver sections stained using Oil Red O in mice fed MFSD or MFFD. Scale bars = 100 µm. Each value represents the mean ± standard error of the mean (*n* = 5–6). Differences between groups were assessed by one-way ANOVA with Tukey’s test for multiple comparisons. ^††^ *p* < 0.01 AAV-CTL (MFFD) vs. AAV-NPGL (MFFD). NPGL, neurosecretory protein GL; AAV-CTL, AAV-based control vector; AAV-NPGL, AAV-based NPGL-precursor gene vector; MFSD, medium-fat/medium-sucrose diet; MFFD, medium-fat/medium-fructose diet.

**Figure 4:**
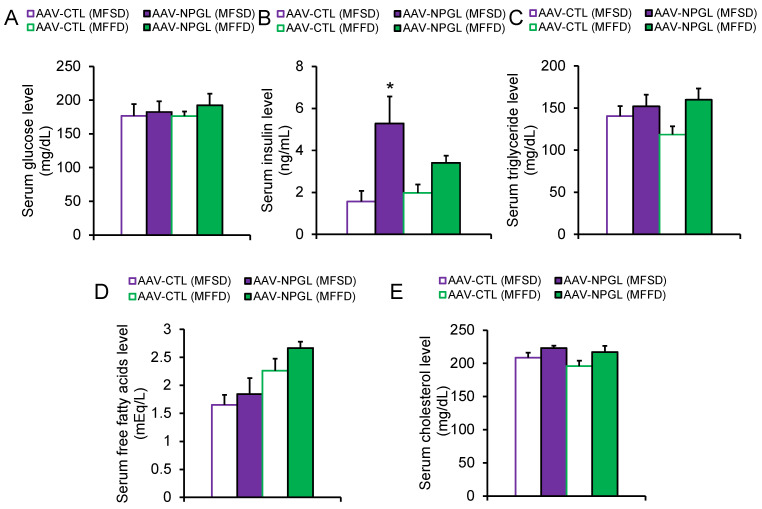
Effects of *Npgl* overexpression on serum parameters. The panels show the data obtained after injection of AAV-CTL or AAV-NPGL into mice fed MFSD or MFFD for 28 days. (**A**) Serum level of glucose. (**B**) Serum level of insulin. (**C**) Serum level of triglyceride. (**D**) Serum level of free fatty acids. (**E**) Serum level of cholesterol. Each value represents the mean ± standard error of the mean (*n* = 5–6). Differences between groups were assessed by one-way ANOVA with Tukey’s test for multiple comparisons. * *p* < 0.05 AAV-CTL (MFSD) vs. AAV-NPGL (MFSD). NPGL, neurosecretory protein GL; AAV-CTL, AAV-based control vector; AAV-NPGL, AAV-based NPGL-precursor gene vector; MFSD, medium-fat/medium-sucrose diet; MFFD, medium-fat/medium-fructose diet.

To determine why *Npgl* overexpression induced an increase in body mass, we measured the masses of adipose tissues, muscles, and several organs (Figure 2 and Figure 3). In mice fed MFSD, the masses of epididymal WAT (eWAT) and perirenal WAT (pWAT) were significantly increased compared to those of control mice. Inguinal WAT (iWAT) and retroperitoneal WAT (rWAT) were slightly affected, but the difference was not significant (Figure 2A). Under MFFD, the masses of iWAT, eWAT, rWAT, and pWAT were increased by *Npgl* overexpression (Figure 2A). Hematoxylin and eosin staining revealed larger adipocytes in the iWAT of mice fed both diets (Figure 2B). In contrast, the mass of the gastrocnemius muscle was not affected by *Npgl*-overexpressing mice under the two dietary conditions (Figure 3A). Subsequently, we measured the masses of peripheral organs. The mass of the liver was increased in mice fed MFFD but not in mice fed MFSD (Figure 3B). Oil Red O staining showed steatosis in the liver of *Npgl*-overexpressing mice (Figure 3C). Measurement of blood glucose, insulin, and lipid levels in *Npgl*-overexpressing mice fed MFSD indicated an increase in circulating insulin levels, but no change was observed in the other serum parameters (Figure 4A–E).

### 2.3. Effects of NPGL-Precursor Gene Overexpression on the mRNA Expression of Lipid Metabolism-Related Genes

**Figure 5:**
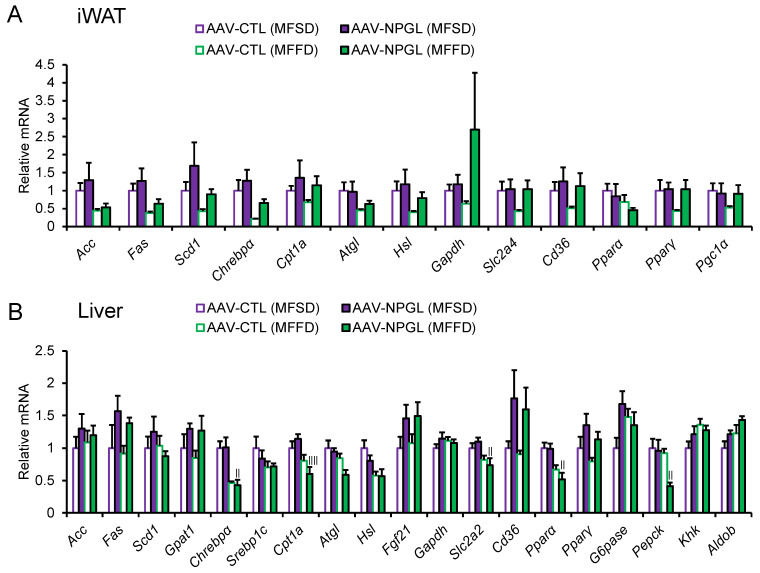
Effects of *Npgl* overexpression on the mRNA expression of lipid metabolism-related genes. The panels show the data obtained after injection of AAV-CTL or AAV-NPGL into mice fed MFSD or MFFD for 28 days. (**A**) mRNA expression in iWAT. (**B**) Hepatic mRNA expression. Each value represents the mean ± standard error of the mean (*n* = 5–6). Differences between groups were assessed by one-way ANOVA with Tukey’s test for multiple comparisons. ^||^ *p* < 0.05, ^||||^ *p* < 0.01 AAV-NPGL (MFSD) vs. AAV-NPGL (MFFD). NPGL, neurosecretory protein GL; AAV-CTL, AAV-based control vector; AAV-NPGL, AAV-based NPGL-precursor gene vector; MFSD, medium-fat/medium-sucrose diet; MFFD, medium-fat/medium-fructose diet; WAT, white adipose tissue; iWAT, inguinal WAT.

After we observed the effect of NPGL on fat accumulation, we measured the mRNA expression of lipid metabolism-related genes using quantitative reverse transcriptase PCR (qRT-PCR) in the iWAT and liver of mice fed MFSD or MFFD. The expression of the following genes was measured: acetyl-CoA carboxylase (*Acc*), fatty acid synthase (*Fas*), stearoyl-CoA desaturase 1 (*Scd1*), and glycerol-3-phosphate acyltransferase 1 (*Gpat1*) as genes encoding lipogenic enzymes; carbohydrate-responsive element-binding protein α (*Chrebpα*) and sterol regulatory element-binding protein 1c (*Srebp1c*) as genes encoding lipogenic transcription factors; carnitine palmitoyltransferase 1a (*Cpt1a*), adipose triglyceride lipase (*Atgl*), hormone-sensitive lipase (*Hsl*), and *Fgf21* as genes encoding lipolytic enzymes; glyceraldehyde-3-phosphate dehydrogenase (*Gapdh*) as a carbohydrate metabolism enzyme-coding gene; solute carrier family 2 member 4 and 2 (*Slc2a4* and *Slc2a2*) as glucose transporter-coding genes; cluster of differentiation 36 (*Cd36*) as a fatty acid transporter-coding gene; peroxisome proliferator-activated receptor α (*Pparα*) and γ (*Pparγ*) as genes encoding lipid-activated transcription factors; peroxisome proliferator-activated receptor γ coactivator 1α (*Pgc1α*) as a thermogenic regulator-coding gene; glucose 6-phosphatase (*G6pase*) and phosphoenolpyruvate carboxykinase (*Pepck*) as genes encoding key rate-limiting enzymes for gluconeogenesis; ketohexokinase (*Khk*) and aldolase B (*Aldob*) as genes encoding key enzymes for fructose metabolism. In the iWAT, one-way ANOVA revealed that there were no changes in mRNA expression following *Npgl* overexpression (Figure 5A). Two-way ANOVA showed that the mRNA expression of *Acc*, *Fas*, and *Chrebpα* was lower in mice fed MFFD than in those fed MFSD (Figure 5A, Table 1). In the liver, the mRNA expression of *Chrebpα*, *Cpt1a*, *Slc2a2*, *Pparα*, and *Pepck* was downregulated in *Npgl*-overexpressing mice fed MFFD compared to those fed MFSD (Figure 5B). qRT-PCR analysis by two-way ANOVA showed that MFFD consumption decreased the mRNA expression of *Chrebpα*, *Cpt1a*, *Atgl*, *Hsl*, *Slc2a2*, *Pparα*, and *Pepck* compared with MFSD consumption, whereas it increased the expression of *Aldob* (Figure 5B, Table 2). The main effect of treatment was observed on the mRNA expression of *Fgf21*, *Cd36*, and *Pparγ* (Figure 5B, Table 2).

## 3. Discussion

Sugar is the root cause of many diseases, such as metabolic syndrome, via dysfunction of systemic energy metabolism. We recently showed that hypothalamic overexpression of *Npgl*, a novel precursor gene encoding a small protein, induces obesity by enhancing the palatability of carbohydrates in rodents [15,20]. However, the difference in lipogenic efficiency of NPGL with different types of sugars remains unclear. In this study, we induced hypothalamic overexpression of *Npgl* and subsequently found potent fat accumulation and liver steatosis in mice fed MFFD relative to those consuming MFSD. The present data imply that NPGL exacerbates fatty liver in fructose-fed mice.

We confirmed the presence of fatty liver in *Npgl*-overexpressing mice fed MFFD by determining the hepatic mass, Oil Red O staining, and upregulation of *Pparγ* mRNA expression. Excess input of lipids over output in the liver cause steatosis as well as systemic lipid accumulation [21]. Liver steatosis is not only caused by de novo lipogenesis but also by the intake of free fatty acids. A previous study reported that a high-fructose diet enhances the release of fatty acids from adipocytes [22]. In addition, lipid oxidation is involved in the output (i.e., lipids released from the liver) [21]. Exposure to fructose suppresses lipid oxidation in the liver [23,24]. In the present data analyzed by two-way ANOVA, the mRNA expression of factors related to lipid oxidation, such as *Cpt1a*, *Atgl*, *Hsl*, and *Pparα* were downregulated in the liver of mice fed MFFD. Notably, one-way ANOVA showed that *Cpt1a* and *Pparα* were downregulated in *Npgl*-overexpressing mice fed MFFD compared to those consuming MFSD. Moreover, two-way ANOVA demonstrated that the mRNA expression of *Cd36*, which is involved in fatty acid intake, was significantly upregulated by *Npgl* overexpression. Hence, we speculate that the synergistic effects of fructose and NPGL exacerbate liver steatosis.

Even though our previous study reported the orexigenic effects of NPGL [15,16,25], the present study emphasized that *Npgl* overexpression does not affect sugar-rich food intake. The endocrine system, including neuropeptides and hormones, regulate nutrient preferences. CRH-positive neurons participate in the selection of high-carbohydrate diets [12]. In addition, FGF21, a hormone secreted by the liver, downregulates sweet-seeking behavior and food intake [13]. Our data indicated that the mRNA expression of *Fgf21* was upregulated in the livers of *Npgl*-overexpressing mice. Thus, it is possible that the orexigenic effects of NPGL were masked by the anorexigenic effects of FGF21 against sugar. Recently, we reported that the effects of NPGL on feeding behavior depend on dietary nutritional composition in rats [19]. Further studies are needed to understand the relationship between the regulation of feeding behavior by NPGL and the nutritional components of different diets.

This study revealed that *Npgl* overexpression increased serum insulin levels in mice fed MFSD, whereas insulin levels hardly increased in mice fed MFFD. Some reports have demonstrated that the effects of sugar on insulin secretion depend on the type of sugar involved. A sucrose-rich diet promotes glucose-induced insulin secretion [26]. Meanwhile, excess intake of high-fructose corn syrup leads to impaired glucose tolerance due to insulin secretion deficiency [27]. We recently reported that *Npgl* overexpression increases circulating insulin levels in mice fed a high-calorie diet, including medium-sucrose [20]. Hence, the effects of NPGL on insulin secretion were disturbed by ingestion of MFFD. Further research is required to elucidate the effects of NPGL on insulin secretion under different dietary components involving sugar.

The present study had several limitations. First, we assessed lipid metabolism in iWAT and liver using qRT-PCR. In iWAT, *Npgl* overexpression had a limited effect on the mRNA expression. Since lipid metabolic factors are controlled at both transcriptional and post-translational levels, NPGL may affect lipid metabolism at the protein level in iWAT. To date, the receptor for NPGL has not been found. Additional studies to measure the enzymatic activities of lipid metabolic factors and to identify the receptor for NPGL will help to understand the lipid metabolic regulation by NPGL. In addition, since previous studies indicate that the lipogenic effects of NPGL are more potent in rodents fed a fat-rich diet [15,20,28], we used MFSD or MFFD, which includes fat as well as sucrose or fructose in this study. However, it is well-known that dietary fat affects energy metabolism [29]. For instance, ChREBP, a key regulator of de novo lipogenesis, is activated in response to glucose and fructose and inactivated by an increase in fatty acids [30,31]. Thus, several studies into low-fat/high-sucrose or high-fructose conditions will enable us to investigate the lipogenic effects of NPGL under different sugar types in detail. Moreover, the present study showed that *Npgl* overexpression had little effect on serum parameters, although it led to excess adiposity in mice fed MFFD. Several reports have demonstrated that the intake of dietary fructose causes metabolic disorders [4,5]. The present data imply that *Npgl* overexpression in the moderate term (i.e., 28 days) maintains a steady metabolic state, although it easily provokes fatty liver in mice fed MFFD. Further studies involving long-term analysis will open new avenues into the relationship between types of sugar consumed and metabolic abnormalities.

In conclusion, we showed that NPGL augments the effects of fructose on lipid accumulation, including fatty liver, using AAV-induced overexpression in mice. This is the first report demonstrating differences in the lipogenic efficiency of NPGL according to the type of sugar consumed. Progress in research surrounding the effects of NPGL on lipid metabolism in several nutritional conditions, including types of sugar or fatty acids, will help us to better understand the relationship between hypothalamic regulation and obesity-related diseases such as nonalcoholic steatohepatitis under different nutritional conditions.

## 4. Materials and Methods

### 4.1. Animals

Male C57BL/6J mice (7 weeks old) were purchased from Nihon SLC (Hamamatsu, Japan) and individually housed under standard conditions (25 ± 1 °C under a 12 h light/12 h dark cycle) with *ad libitum* access to water and MFSD (32% of calories from fat, 20% of calories from sucrose, D14050401; Research Diets, New Brunswick, NJ, USA) or MFFD (32% of calories from fat, 20% of calories from fructose, D19061101; Research Diets). Nutritional compositions are shown in Table 3. Animal surgery was conducted under isoflurane anesthesia. All animal experiments were performed according to the Guide for the Care and Use of Laboratory Animals prepared by Hiroshima University (Higashi-Hiroshima, Japan), and these procedures were approved by the Institutional Animal Care and Use Committee of Hiroshima University (permit numbers: C19-8, 30 August 2019; C21-1, 19 April 2021).

### 4.2. Production of AAV-Based Vectors

AAV-based vectors were generated following a previously reported method [20,32]. In this study, the primers for mouse NPGL were 5’-CGATCGATACCATGGCTGATCCTGGGC-3’ for the sense primer and 5’-CGGAATTCTTATTTTCTCTTTACTTCCAGC-3’ for the antisense primer. AAV-based vectors were prepared at a concentration of 1 × 10^9^ particles/µL and stored at −80 °C until use.

### 4.3. Stereotaxic Surgery

*Npgl* overexpression was conducted as previously described [20,32]. Mice were bilaterally injected with 0.5 µL/site (5.0 × 10^8^ particles/site) of AAV-based vectors (AAV-NPGL or AAV-CTL) using a Neuros Syringe (7001 KH; Hamilton, Reno, NV, USA) into the mediobasal hypothalamic region with the coordinates 2.2 mm caudal to the bregma, 0.25 mm lateral to the midline, and 5.8 mm ventral to the skull surface. *Npgl* overexpression was maintained for 28 days in mice fed with MFSD or MFFD. *Npgl* overexpression was confirmed by qRT-PCR at the endpoint (Figure S1).

### 4.4. Measurement of Body Mass, Food Intake, and Body Composition

The mice were divided into two groups according to their diet (MFSD or MFFD). Food intake and body mass were measured at the beginning of the light period (9:00). Food efficiency (g/kcal) was calculated as body mass gain (g)/cumulative food intake (kcal) [33]. A total of 28 days after stereotaxic surgery, the mice were decapitated between 13:00 and 15:00. The mediobasal hypothalamus, adipose tissues, organs, and skeletal muscles were collected, weighed, and frozen in liquid nitrogen. Blood was collected at the same time as the mice were sacrificed.

### 4.5. qRT-PCR

Total RNA was extracted using QIAzol lysis reagent (QIAGEN, Venlo, Netherlands) for iWAT or TRIzol reagent (Life Technologies, Carlsbad, CA, USA) for hepatic tissue and the mediobasal hypothalamus, according to the manufacturer’s instructions. First-strand cDNA was synthesized from total RNA using a ReverTra Ace kit (TOYOBO, Osaka, Japan).

Sequences of primers used in this study are listed in Table 4. PCR amplifications were conducted with THUNDERBIRD SYBR qPCR Mix (TOYOBO) using the following conditions: 95 °C for 20 s, followed by 40 cycles each consisting of 95 °C for 3 s and 60 °C for 30 s. The PCR products in each cycle were monitored using a Bio-Rad CFX Connect (Bio-Rad Laboratories, Hercules, CA, USA). Relative quantification of each gene was determined by the 2^−ΔΔCt^ method using ribosomal protein S18 (*Rps18*) for iWAT or β-actin (*Actb*) for the liver and mediobasal hypothalamus as an internal control [34]. The expression of internal control genes was verified to be stable across the experimental groups.

### 4.6. Hematoxylin and Eosin Staining

iWAT was soaked in 4% paraformaldehyde at the endpoint of *Npgl* overexpression, embedded in paraffin, and sectioned to a thickness of 8 µm using a microtome. The sections were then air-dried and deparaffinized in a graded alcohol series. Nuclei and cytoplasm were stained with hematoxylin and eosin (5 min for each stain), and the sections were washed with tap water. After dehydration in a graded alcohol series and clearing with xylene, the sections were mounted on slides and examined under a microscope.

### 4.7. Oil Red O Staining

To detect fat accumulation in the liver, hepatic tissue was fixed in 4% paraformaldehyde and sliced into 10-μm-thick sections. Sections were air-dried, rinsed with 60% isopropanol, stained with Oil Red O solution for 15 min at 37 °C, and rinsed with 60% isopropanol. Nuclei were counterstained with hematoxylin for 5 min, and the sections were then washed with tap water. Coverslips were applied using an aqueous mounting medium, and microscopic examination was performed using a microscope.

### 4.8. Serum Biochemical Analysis

Serum levels of glucose, insulin, and lipids were measured using appropriate equipment, reagents, and kits. Glucose content was measured using a GLUCOCARD G+ meter (Arkray, Kyoto, Japan). The LBIS Insulin-mouse T ELISA kit (Shibayagi, Gunma, Japan) was used to measure insulin levels. The NEFA C-Test Wako (Wako Pure Chemical Industries, Osaka, Japan) was used to measure free fatty acid abundance. Triglyceride E-Test Wako (Wako Pure Chemical Industries) was used to measure triglyceride levels, and the Cholesterol E-Test Wako (Wako Pure Chemical Industries) was used to assess cholesterol content.

### 4.9. Statistical Analysis

To evaluate the effects of *Npgl* overexpression in mice fed two diets, one-way ANOVA with Tukey’s test for multiple comparisons was performed. Two-way ANOVA with repeated measures followed by Bonferroni’s test for multiple comparisons was used to assess the main effects of groups (AAV-CTL (MFSD), AAV-NPGL (MFSD), AAV-CTL (MFFD), AAV-NPGL (MFFD)) and time, and the effects of interactions between the groups and time. If significant effects of interactions between groups and time were observed, the results of one-way ANOVA with Tukey’s test for multiple comparisons in the same period were shown instead of Bonferroni’s test of the main effects of groups. To determine the effects of different treatments (injection of AAV-CTL or AAV-NPGL) and different diets (MFSD or MFFD) in the qRT-PCR analysis, two-way ANOVA was conducted (Table 1 and Table 2). Statistical significance was set at *p* < 0.05.

## Figures and Tables

**Table 1:** Results of two-way ANOVA on mRNA expression in iWAT. Bold font indicates statistical significance.

Gene	Treatment		Diet		Interaction	
*Acc*	F(1, 19) = 0.39	0.538	F(1, 19) = 4.55	**<0.05**	F(1, 19) = 0.10	0.755
*Fas*	F(1, 19) = 1.20	0.287	F(1, 19) = 6.96	**<0.05**	F(1, 19) = 0.001	0.979
*Scd1*	F(1, 19) = 2.02	0.172	F(1, 19) = 2.78	0.112	F(1, 19) = 0.07	0.795
*Chrebp* *α*	F(1, 19) = 2.16	0.158	F(1, 19) = 8.31	**<0.01**	F(1, 19) = 0.12	0.737
*Cpt1a*	F(1, 19) = 1.70	0.208	F(1, 19) = 0.70	0.415	F(1, 19) = 0.03	0.853
*Atgl*	F(1, 19) = 0.11	0.740	F(1, 19) = 4.35	0.051	F(1, 19) = 0.23	0.634
*Hsl*	F(1, 19) = 1.01	0.327	F(1, 19) = 3.03	0.098	F(1, 19) = 0.14	0.710
*Gapdh*	F(1, 19) = 2.05	0.168	F(1, 19) = 0.55	0.486	F(1, 19) = 1.45	0.243
*Slc2a4*	F(1, 19) = 1.85	0.189	F(1, 19) = 1.45	0.244	F(1, 19) = 1.40	0.251
*Cd36*	F(1, 19) = 1.88	0.186	F(1, 19) = 0.95	0.341	F(1, 19) = 0.32	0.576
*Ppar* *α*	F(1, 19) = 0.58	0.455	F(1, 19) = 1.87	0.187	F(1, 19) = 0.15	0.906
*Ppar* *γ*	F(1, 19) = 1.94	0.180	F(1, 19) = 1.47	0.240	F(1, 19) = 1.49	0.237
*Pgc1* *α*	F(1, 19) = 0.41	0.531	F(1, 19) = 1.04	0.321	F(1, 19) = 1.00	0.329

**Table 2:** Results of two-way ANOVA on mRNA expression in liver. Bold font indicates statistical significance.

Gene	Treatment		Diet		Interaction	
*Acc*	F(1, 19) = 0.96	0.339	F(1, 19) = 0.01	0.972	F(1, 19) = 0.22	0.644
*Fas*	F(1, 19) = 3.94	0.062	F(1, 19) = 0.27	0.606	F(1, 19) = 0.04	0.848
*Scd1*	F(1, 19) = 0.05	0.818	F(1, 19) = 0.78	0.389	F(1, 19) = 1.15	0.297
*Gpat1*	F(1, 19) = 3.74	0.068	F(1, 19) = 0.23	0.638	F(1, 19) = 0.11	0.739
*Chrebp* *α*	F(1, 19) = 0.01	0.913	F(1, 19) = 23.54	**<0.005**	F(1, 19) = 0.04	0.841
*Srebp1c*	F(1, 19) = 0.26	0.613	F(1, 19) = 2.29	0.147	F(1, 19) = 0.41	0.532
*Cpt1a*	F(1, 19) = 0.10	0.758	F(1, 19) = 12.49	**<0.005**	F(1, 19) = 2.76	0.113
*Atgl*	F(1, 19) = 2.76	0.113	F(1, 19) = 7.72	**<0.05**	F(1, 19) = 1.20	0.286
*Hsl*	F(1, 19) = 0.88	0.361	F(1, 19) = 9.68	**<0.01**	F(1, 19) = 0.78	0.389
*Fgf21*	F(1, 19) = 4.62	**<0.05**	F(1, 19) = 0.08	0.785	F(1, 19) = 0.01	0.922
*Gapdh*	F(1, 19) = 0.58	0.454	F(1, 19) = 0.09	0.764	F(1, 19) = 1.40	0.252
*Slc2a2*	F(1, 19) = 0.01	0.935	F(1, 19) = 9.61	**<0.01**	F(1, 19) = 1.05	0.319
*Cd36*	F(1, 19) = 5.64	**<0.05**	F(1, 19) = 0.19	0.671	F(1, 19) = 0.02	0.902
*Ppar* *α*	F(1, 19) = 0.78	0.389	F(1, 19) = 19.13	**<0.005**	F(1, 19) = 0.59	0.451
*Ppar* *γ*	F(1, 19) = 4.87	**<0.05**	F(1, 19) = 1.85	0.190	F(1, 19) = 0.002	0.961
*G6pase*	F(1, 19) = 2.09	0.164	F(1, 19) = 0.15	0.704	F(1, 19) = 4.48	<0.05
*Pepck*	F(1, 19) = 4.22	0.054	F(1, 19) = 5.42	**<0.05**	F(1, 19) = 3.07	0.096
*Khk*	F(1, 19) = 0.34	0.565	F(1, 19) = 3.60	0.073	F(1, 19) = 1.78	0.199
*Aldob*	F(1, 19) = 4.01	0.060	F(1, 19) = 4.44	**<0.05**	F(1, 19) = 0.001	0.977

**Table 3:** The artificial diets used in this study.

Diet	Protein (kcal%)	Carbohydrate (kcal%)	Fat (kcal%)	kcal/g
		Sucrose/Fructose (kcal%)		
Medium-fat/medium-sucrose diet (MFSD)	20	48	32	4.4
		19/0		
Medium-fat/medium-fructose diet (MFFD)	20	48	32	4.4
		0/19		

**Table 4:** Sequences of oligonucleotide primers for qRT-PCR.

Gene	Sense Primer (5′ to 3′)	Antisense Primer (5′ to 3′)
*Acc*	TCCGCACTGACTGTAACCACAT	TGCTCCGCACAGATTCTTCA
*Fas*	AGGGGTCGACCTGGTCCTCA	GCCATGCCCAGAGGGTGGTT
*Scd1*	CTGTACGGGATCATACTGGTTC	GCCGTGCCTTGTAAGTTCTG
*Gpat1*	TCATCCAGTATGGCATTCTCACA	GCAAGGCCAGGACTGACATC
*Chrebp* *α*	CGACACTCACCCACCTCTTC	TTGTTCAGCCGGATCTTGTC
*Srebp1c*	GGAGCCATGGATTGCACATT	GGCCCGGGAAGTCACTGT
*Cpt1a*	CCTGGGCATGATTGCAAAG	GGACGCCACTCACGATGTT
*Atgl*	AACACCAGCATCCAGTTCAA	GGTTCAGTAGGCCATTCCTC
*Hsl*	GCTGGGCTGTCAAGCACTGT	GTAACTGGGTAGGCTGCCAT
*Fgf21*	CCTCTAGGTTTCTTTGCCAACAG	AAGCTGCAGGCCTCAGGAT
*Gapdh*	AAGGTCATCCCAGAGCTGAA	CTGCTTCACCACCTTCTTGA
*Slc2a2*	GGCTAATTTCAGGACTGGTT	TTTCTTTGCCCTGACTTCCT
*Slc2a4*	GTAACTTCATTGTCGGCATGG	AGCTGAGATCTGGTCAAACG
*Cd36*	TCCTCTGACATTTGCAGGTCTATC	AAAGGCATTGGCTGGAAGAA
*Ppar* *α*	TCGAATATGTGGGGACAAGG	GACAGGCACTTGTGAAAACG
*Ppar* *γ*	GCCCTTTGGTGACTTTATGGA	GCAGCAGGTTGTCTTGGATG
*Pgc1* *α*	GCAACATGCTCAAGCCAAAC	TGCAGTTCCAGAGAGTTCCA
*G6pase*	ACTGTGGGCATCAATCTCCTC	CGGGACAGACAGACGTTCAGC
*Pepck*	GTGCTGGAGTGGATGTTCGG	CTGGCTGATTCTCTGTTTCAGG
*Khk*	CCTGCCAGATGTGTCTGCTA	TGCAGCATCTTCACCTGTTC
*Aldob*	AGAGGATGGAGAAGGGCATT	ATGCAGGATCCCTCAACAAG
*Npgl*	TATGTAGACTGTGTCCTCTC	TCTAAGGAGCTGAGAATATGCA
*Rps18*	CCTGAGAAGTTCCAGCACAT	TTCTCCAGCCCTCTTGGTG
*Actb*	GGCACCACACCTTCTACAAT	AGGTCTCAAACATGATCTGG

## Data Availability

The raw data supporting the findings of this manuscript will be made available by the corresponding author, K.U., to any qualified researchers upon reasonable request.

## Supplementary Materials

The following supporting information can be downloaded at: https://www.mdpi.com/article/10.3390/ijms23042071/s1.
