# Supplementary material for: Neurosecretory Protein GL Accelerates Liver Steatosis in Mice Fed Medium-Fat/Medium-Fructose Diet
Source: Int J Mol Sci. 2022 Feb 13;23(4):2071. doi: 10.3390/ijms23042071 (PMC8876799; doi:10.3390/ijms23042071)
Supplement: Supplementary file 1 [file ijms-23-02071-s001.zip › ijms-1588824-supplementary.pdf]

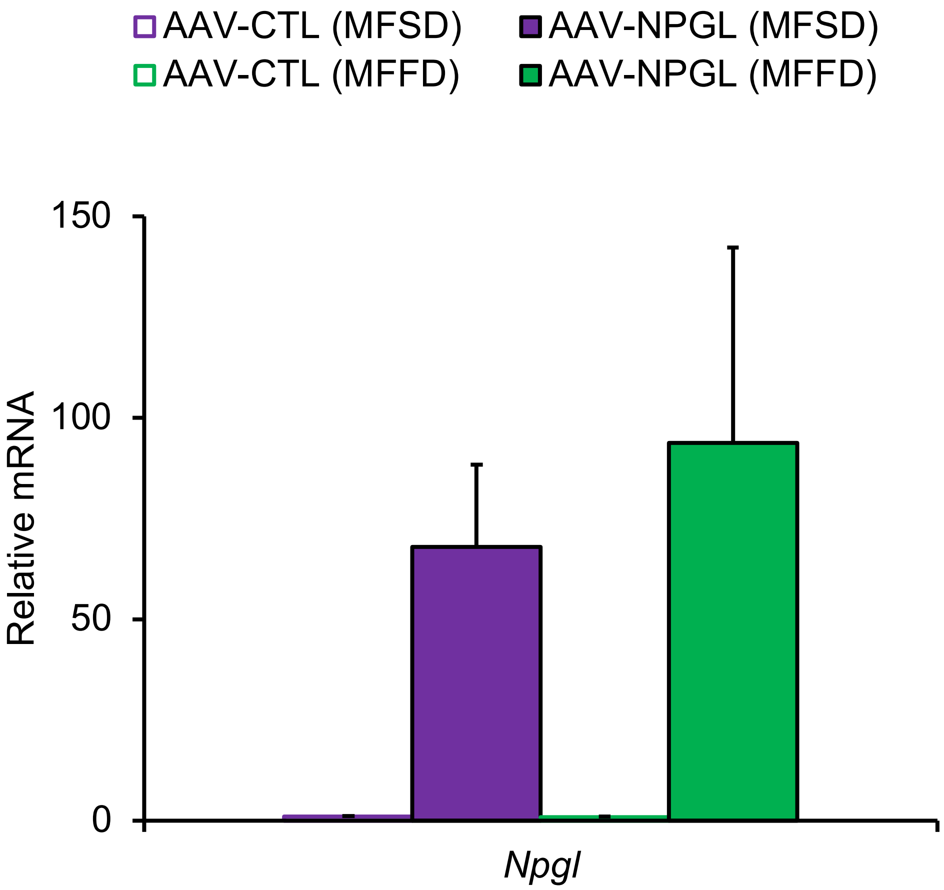

Figure S1. mRNA expression level of *Npgl* in the mediobasal hypothalamus at the endpoint of *Npgl* overexpression. Each value represents the mean ± standard error of the mean (n = 5–6). Differences between groups were assessed by two-way ANOVA (Treatment: $p < 0.01$, Diet: $p = 0.627$, Interaction: $p = 0.625$). NPGL, neurosecretory protein GL; AAV-CTL, AAV-based control vector; AAV-NPGL, AAV-based NPGL-precursor gene vector; MFSD, medium-fat/medium-sucrose diet; MFFD, medium-fat/medium-fructose diet.
